# The effect of immersion on sense of presence and affect when experiencing an educational scenario in virtual reality: A randomized controlled study

**DOI:** 10.1016/j.heliyon.2023.e17196

**Published:** 2023-06-12

**Authors:** Tuva Fjærtoft Lønne, Håvard R. Karlsen, Eva Langvik, Ingvild Saksvik-Lehouillier

**Affiliations:** Department of Psychology, Norwegian University of Science and Technology, 7491, Trondheim, Norway

**Keywords:** Learning, Virtual reality, Immersion, Sense of presence, Affect, Soft skills

## Abstract

Virtual reality (VR) technology has been used to learn skills for decades. While no standardized measure exists for learning outcomes in VR training, commonly explored outcomes are immersion, sense of presence and emotions.

**Methods:**

In this paper, the objective was to investigate these outcomes in two VR conditions, immersive and desktop in a randomized controlled trial with a parallel design. The sample consisted of 134 university students (70 women, mean age 23 years*, SD* = 2.99). These were randomized using a covariate-adaptive randomization procedure based on stratification by gender into two interventions; play out a VR scenario in either desktop (control group) or immersive VR (intervention group). The setting was a university lab.

**Results:**

There was a significant within subject effect for positive affect and a significant between-group effect for the immersive compared to desktop VR groups. Positive affect was reduced after interacting with the VR scenario in both the immersive and desktop versions, however, positive affect was overall higher in the immersive, compared to the desktop version. The results show higher scores for sense of presence (*d* = 0.90, *p* < 0.001) and positive affect pre- and post-scenario in the immersive VR condition (*d* = 0.42, *p* = 0.017 and *d* = 0.54, *p* = 0.002) compared to the desktop condition.

**Conclusion:**

Immersive VR may be beneficial in higher education as it promotes high levels of sense of presence as well as positive emotions. When it comes to changing the immediate emotions of the students, type of VR does not seem to matter. The project was funded by the Norwegian Directorate for Higher Education and Skills.

## Highlights

• Positive affect was reduced after interacting with the VR scenario in both the immersive and desktop versions
• Positive affect and sense of presence were overall higher in the immersive, compared to the desktop version.
• Because of this, learning outcomes may be higher using immersive VR compared to desktop VR, but this needs to be examined in future studies

## 1 Introduction

There is an increasing use of technology in higher education across most educational fields, gradually replacing traditional face-to-face learning [1]. This is not only due to the new experiences gained by the COVID-19 pandemic where many were forced to use more technology in their education [2], but also because technology now can offer many benefits for teaching in terms of for example possibilities of individualized teaching, improved feedback and better alignment of learning goals [3]. One of these emerging educational technologies is the immersive head-mounted displays (HMD) used to explore different virtual realities (VR) [4]. Immersive VR can be seen as a high-end user interface involving real-time simulations of an environment that users can explore and interact with through multiple senses [5]. *System immersion* is concerned with the objective property of the system, describing to which extent a VR system can support natural sensorimotor contingencies for perception [6]. Sense of *presence* is the subjective experience of being in a place or in an environment, even if one is physically present in another environment [7].

Immersion and presence are positively related to learning outcomes [4,[8], [9], [10]]. However, there is a lack of randomized controlled trials with high enough sample size to examine the effect of immersive VR on variables related to learning. Such studies are challenging, as the variables are difficult to define, and the study requires a high control, while not compromising the ecological validity. Previous studies have usually included between 20 and 50 participants [4], which may not enough to detect real effects. Evaluation of technological applications, and VR in particular is mainly performed in experimental settings, and not as a part of the actual education [11]. Consequently, we have yet to determine how beneficial immersive VR is compared to desktop VR for students in higher education.

Immersive VR technology is increasingly used in education and training [4,12,13] and several higher education institutions have already implemented immersive VR [14]. Immersive VR can make it easier for students to understand complex concepts through visual understanding [4]. Studies comparing immersive VR to desktop VR within learning situations have found that high system immersion predicts positive emotions and positive cognitive evaluation of a task [10] and increase knowledge about a specific topic [15]. However, some studies have found negative or neutral results of immersive VR compared to desktop VR [16,17]. Few of the studies on these field are randomized studies with large experimental groups comparing differences between immersive VR and other educational technologies. This question the overall contribution and the knowledge of the field.

Research also support that the use of immersive VR may not directly increase learning [18], but affect learning through other more indirect processes, as sense of presence [9] or affective processes like intrinsic motivation and engagement [19]. Across studies on VRs, sense of presence has an important role in the elicitation of emotional arousal and sense of realism in the virtual environment [20]. Sense of presence predicts higher emotional arousal [21]. Emotions are often seen as specific states within the umbrella term affect [22]. Emotions have a great influence on multiple cognitive processes such as attention [23], reasoning [24], memory [25], and learning [26]. Emotions have a particularly large effect on attention as well as motivating behaviour and action, which is closely linked to the learning process [27]. Learners seem to remember emotional experiences more accurately and vividly compared to non-emotional content, an effect that lasts over time [1].

Immersive VR may facilitate learning through positive emotions [19,28,29] due to the effect of enjoyment on student performance [e.g. 30, 31]. Moreover, immersive VR may increase motivation, engagement, and interest amongst students [32,33]. Even in studies where immersive VR was shown to have a worse effect on learning than less immersive learning methods, the immersive VR condition was more motivating than the control condition [4]. Even though some studies show a positive effect on emotion when using immersive VR in learning, there is limited evidence on what specific affective value immersive VR have [19] partly due to lack of randomized controlled trials in actual educational settings.

In the past, immersive VR has mainly been used to train specific skills in situations that would be dangerous or demanding to simulate physically [14]. In more recent times there has been a great focus on exploring how immersive VR can be used in teaching soft skills, that is for example social- and interpersonal skills, and several big companies use immersive VR to train their employees in such skills [14,34]. The importance of soft skills globally across university educations are headlined as essential as they affect personal development, communication and interpersonal relations [35].

The benefit of using simulation based techniques to teach soft skills are many, for example the ability to practice real-life communication and decision making practices without potentially harming real patients or students [36]. Virtual reality makes it possible to fully immerse an individual in an affective scenario [37]. Compared to desktop VR, immersive VR may have the potential to immerse the user in such a way that an interpersonal first-person perspective can feel realistic enough to experience the events in the relation as real [38]. Being able to practice soft skills in a realistic environment can improve the individual’s interpersonal relations [39,40].

One of the most established soft skills to train in immersive VR is speaking in front of other people [41]. Speaking in front of people in immersive VR seem to affect the same psychological processes, e.g., increased heart rate, as in real life [42]. Although immersive VR is emerging in the field of soft skills, there are still limited studies addressing these skills. Such studies often show inconclusive results [43] as it is proven hard to measure the results of soft skills training, and to objectify them [44,45]. E.g. to objectively test whether someone has learned to communicate better, or handle critique better is time consuming and hard to quantify. Given the strong role of affect and presence in immersive VR, and their relation to motivation, cognition and learning shown above, it may be more fruitful to measure the role of these in a VR scenario, than to attempt measuring effects on soft skills directly. This can be done as a basis for later research investigating more longitudinal effects of immersive VR on specific soft skills.

### 1.1 Objectives

The objective of the present study was to examine positive affect and negative affect in an immersive VR scenario relevant for soft skill training compared to desktop VR. As sense of presence is important for the immersive VR experience, we also aim to confirm the previously reported tendency of experiencing higher sense of presence in immersive compared to desktop VR. This was assessed in a randomized control trial used in a real educational setting, with high control and a large number of participants.

The hypotheses are as follows:

H1 The level of positive affect will be higher after playing through a virtual scenario in immersive VR than in desktop VR.

H2 The changes in positive affect after playing through a virtual scenario in the immersive VR group will differ from the changes in the desktop VR group.

H3 The changes in negative affect after playing through a virtual scenario in the immersive VR group will differ from the changes in the desktop VR group.

H4 There will be a higher sense of presence in immersive VR than in desktop VR.

## 2 Methods

### 2.1 Design

We used the CONSORT statement to report the present study but did not preregister the study in a trial register. To test the hypotheses a randomized controlled trial was designed. Prior to the present study, a pilot study was run. This was to make sure that the experiment would run smoothly, that the instructions were clear, that the design worked as planned, and to identify other experimental challenges. As a result of this pilot study, multiple factors were adjusted such as the translation of the questionnaire, final adjustments of the interactive video to exclude bugs, the instructions given to the participants, as well as the design of the study. The original design was a crossover-design where all participants were tested in both the immersive VR and desktop VR condition. Half of them started in the immersive VR, and half in the desktop VR. All participants reported that they got bored when playing through the scenario multiple times in each group which compromised the truthfulness of the answers on the questionnaire. Resultingly, the design of the study was changed. Thus, the final study consisted of two independent groups, testing two independent setups to ensure that none of the participants had played through the scenario before, making it a novel experience for everyone. Thus, a parallel design was used.

A mixed 2 × 2 design consisting of two (immersive VR and desktop VR) condition between-subjects and two timepoints (before and after) was implemented. The participants were randomly assigned to play through an interactive scenario in VR displaying a work-related conflict relevant for soft skills in one of the two conditions. Positive affect and negative affect were measured before and after playing through the scenario. User experience, including sense of presence was measured after the scenario.

### 2.2 Participants

Prior to the data collection, a power analysis was performed to compute the required sample size. G*Power v.3.1.9.2 was used for this task, with the following parameters: ANOVA, repeated measures, within-between interactions. For effect size, we used direct input, f(v) 0.25 the alpha level 0.05, and the power to 0.80. The effect size input was selected using the function in G*Power that bases the calculation on recommendations from Cohen [46]. The power analysis tool returned a recommended sample size of 126 participants. Participants were recruited based on this, randomly assigned to one of the groups, matched by gender, to ensure that the sample was representative of the population.

Participants were recruited amongst the students at the local University. Inclusion criteria were that they had to be students at the university and had to be between 19 and 40 years of age. Participant recruitment strategies used included announcements in lectures, snowballing methods where participants themselves informed peers, announcements on the teaching and learning platform Blackboard, posters in the hallways, directly approaching candidates at the university, and announcements on social media. As a reward for participating in the study, five randomly selected participants across the groups got a gift card of 200 NOK. The participants in the desktop VR group also got free pizza after participation.

A total of 134 individuals were recruited and assessed for eligibility, all met the inclusion criteria, all gave their informed consent and all completed the entire study (*N* = 134, 70 female and 63 male, one participant reported gender as “other”). None were excluded, did not receive allocated intervention nor were lost to follow up (see flow diagram). None of them had participated in the pilot study. The ages ranged from 19 to 40 years (*M* = 23*, SD* = 2.99). Using a covariate-adaptive randomization procedure [47] based on stratification by gender the participants were assigned into two groups; To play through the scenario in either immersive VR (intervention group) (*n* = 69, 34 female) or desktop VR (control group) (*n* = 65, 36 female). The randomization was performed by one of the study administrators as the participants signed up for the study and was evaluated for eligibility. The participants first indicated what day and time they could come in for testing. This study administrator then assigned participants into the control group or intervention group by alternating between the groups by every new eligible participant, at the same time also bearing in mind the preferred day and time and the gender distribution to obtain an even gender distribution in both groups. The participants were blinded to the intervention before taking part in it, but discovered if they had been assigned to the control or intervention group by themselves immediately after starting the trial.

### 2.3 Procedure

The study was carried out at the university campus, in small classrooms. The trial was administered by one of the authors with the help of two student assistants during the fall of 2021, from the 20th of September to the 10th of November. In the immersive VR condition, the participants were tested in groups of maximum three people at a time. They were each assigned to a setup with an HMD, a protective face mask, a computer, two position sensors, and one hand-controller. In the desktop VR condition, the participants were tested in groups of maximum fourteen people at a time. The participants were tested in a room with three rows of computers prepared for the experiment. The computers were stationed with a 1-m gap between them. Each of the participants were assigned to a setup including a computer, a computer-mouse, and an audio headset.

Before starting the experiment both groups were given oral information about consent, the equipment, the questionnaire, the video, that the experiment was expected to last for approximately 340 min, and that they could end the experiment at any point without any consequences. This information was also given in writing before the start of the questionnaire. All participants signed a letter of consent. After been given the information, the participants answered the first part of the questionnaire: the demographics and the Positive and Negative Affect Schedule (PANAS [48]) pre.

In the immersive VR condition, the participants were asked to stand after answering the PANAS pre before an experimenter who instructed them on how to put on and adjust the HMD. They were instructed on how to put on the protective face mask, the HMD itself, adjust the head straps, the inter-pupillary distance, the speakers, and the hand-controller. They continued to stand when playing through the scenario.

When all the participants were done with the first part of the questionnaire, they were given instructions that there were to play through the scenario three times, trying out different in-game alternatives, and that when they were done, they were to continue to the second part of the questionnaire. After playing through the scenario three times, the participants of both groups found their way to the questionnaires on the computer themselves and answered the second part of the questionnaire consisting of PANAS [48] post and the user experience questionnaire. Both groups answered the questionnaires on the computers using a web-based survey builder. This was the last part of the experiment.

Prior to the data collection, the ethical and data storage aspects of the study were approved by the Norwegian Centre for Research Data (application number: 693393).

### 2.4 Materials

#### 2.4.1 Software

**Fig. 1:**
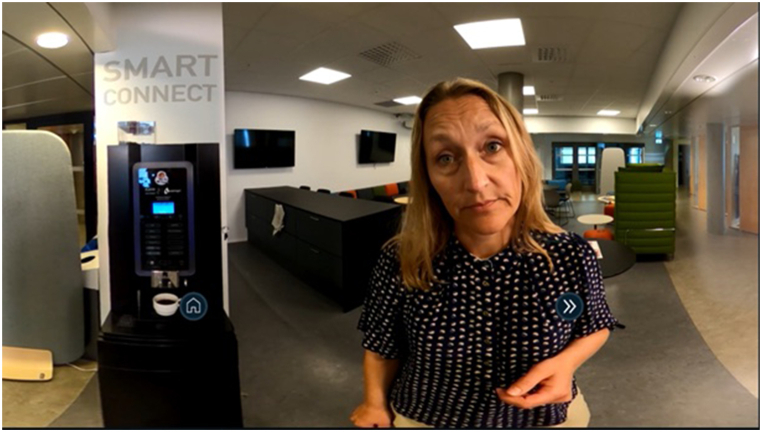
Screen capture of the video illustrating the virtual scenario.

The interactive scenario used in the experiment was produced for use in a lecture in work and organisational psychology and was recorded using a 360°-camera, recording live action. The scenario had a duration of approximately 2 min and showed an interactive dialogue between an employee, acted out by a male actor (named “Olaf”), but played in a first-person perspective by the participant, and the employee’s manager (named “Berit”), acted out by a female actor. The scene was a potential conflict situation, where the manager comes close up to the player and after a short session of small talk comment that the worker’s last decision was made too quickly. A set of optional dialogue choices and their consequences follows in an interactive video. The player can choose three different sets of answers; a) asking what Berit means (avoiding), b) say that it was Berits' own fault, she should have been clearer and is always like this (aggressive/person oriented) and c) admit to being too quick (constructive/solution oriented). Several new dialogues follows until the player once again get to choose what to answer, with similar choice options. The participants played through the scenario as the employee, not seeing the body of their character in the video but answering with the actor’s voice. See Fig. 1 for a screen capture of the video illustrating the scenario.

When playing through the scenario, the participants themselves chose, at multiple times, amongst different pre-recorded alternatives, which response to give to the manager. The manager’s responses depended on which alternatives were chosen. The reaction led the dialogue to end in either a positive manner, a neutral promise of a follow up conversation with the manger, or in a conflict. Thus, all participants played through three different versions of the scenario resulting in both negative and positive outcomes. These points of interaction happened at three different points in the scenario.

#### 2.4.2 Hardware

Even though the two VR conditions were made to be as similar as possible, there were some differences between the two conditions. Participants had full freedom of camera movement in the immersive VR conditions, meaning they had three degrees of freedom. They were not able to move around in the VR condition. In the desktop VR condition, participants had no control over the camera. The camera was mostly fixed in one direction, apart from in the beginning where a small camera pan occurred from a coffee machine to the person speaking.

The group in the immersive VR version used an immersive VR HMD (Oculus Rift) connected to a compatible laptop with two position sensors. The 470 g HMD consists of an OLED display with a resolution of 2160 × 1200 pixels (1080 × 1200 pixels per eye) and a field of view of 110°, as well as integrated “3D audio” headphones. In addition, they had a controller in their hand to choose a response in the scenario. Participants in the desktop VR group played through the scenario on a standard 23-inch stationary computer screen with a resolution of 1920 × 1080 pixels accompanied by on-ear headphones. A computer mouse was used to choose a response in the scenario.

### 2.5 Measures of emotions and user experience (outcomes)

To measure the participant’s affect and subjective user experience of the VR, a questionnaire was developed based on existing questionnaires [49]. The participants answered the first part (consisting of demographics and PANAS [48]) before playing through the scenario, and the second/final part (consisting of PANAS and user experience) directly after playing through the scenario.

#### 2.5.1 Affect

The Positive and Negative Affect Schedule (PANAS) was used to measure the participant’s affect both before and after playing through the scenario [48]. A Norwegian translation of PANAS was used to measure to which extent the participants experienced 20 different affects on a 5-point Likert scale ranging from “not at all” to “very much”. The scale consists of 10 questions measuring positive affect (PA) (interested, alert, attentive, excited, enthusiastic, inspired, proud, determined, strong, active) and 10 questions measuring negative affect (NA) (distressed, upset, guilty, hostile, irritable, nervous, jittery, scared, afraid). Omega (ω) was used as it is generally viewed as superior measure compared to alfa [50]. In the present study the internal consistency was high (ω = 00.81 PA before intervention) to 0.87 (PA after intervention) and ω = 00.83 (NA before intervention) to 0.87 (NA after intervention). The questionnaire has been used previously to measure affect in virtual environments [e.g. 51]. The first PANAS (PANAS pre) was administrated directly before playing through the scenario, and the second one (PANAS post) directly after playing through the scenario in both conditions. There were some missing data on PANAS and only participants who had answered at least 8 out of 10 affect questions were included in the analyses.

#### 2.5.2 Sense of presence

System immersion is defined as an objective measure. However, there is no established way to measure such system immersion other than classifying the different VRs as immersive or not according to objective properties. Visual immersion is a prominent part of the system immersion and consists of the components: the size of the field of view of the user, the size of the field of view that the user can orientate in, screen size, screen resolution, stereoscopy, reproduction based on head movement, realism of light, frame rate and refresh rate. Thus, different VR technologies have different measurable degrees of immersion [12]. For example, an immersive VR HMD has a larger immersion than a mobile screen or a standard desktop display. In the present study, the “measure” of system immersion reflects this and is incorporated in the different VR-technologies, one classified as high-immersive (immersive VR) and one classified as non-immersive (desktop VR) [52].

To measure the participants' subjective user experience in the virtual realities, a questionnaire was used consisting of a collection of questions from different well-known questionnaires such as “the presence questionnaire” [49]. Participants answered the questions after answering the PANAS post. The questionnaire consisted of 41 questions with answers on a 10-point Likert scale, as well as three open ended questions where the participants answered through self-produced text. The full questionnaire consisted of multiple subscales to measure the user experience. In this study the subscale “sense of presence” of the questionnaire are used. The questionnaire measures sense of presence subjectively.

**Fig. 2:**
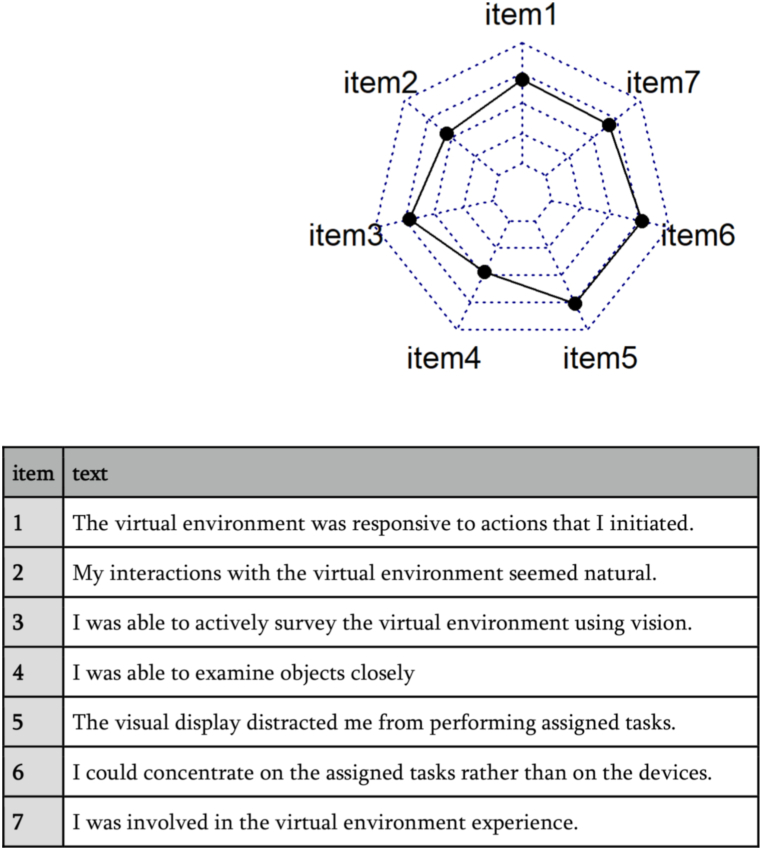
Spider plots showing mean scores on presence items.

Seven of the questions belonged to the subscale sense of presence, e.g. “My interactions with the virtual environment seemed natural”, and “I was able to actively survey the virtual environment using vision”. These items measured how real the participants experienced the scenario to be, if they felt that the experience of being in a conflict with the manager “Berit” felt like a real and natural experience that they could actively engage in. The questionnaire was translated to Norwegian by the first author prior to the experiment to fit the first language of the participants, but no other modifications of the questions were made. See Fig. 2 for a spider plot showing the various presence items. When examining the relationship between the items, we observed that one item (item 5 “The visual display distracted me from performing the assigned tasks) had negative, although very small correlation with two of the other items r −0.02) This was the only reversed item of the instrument. As omega cannot be calculated with negative values, we removed this item, and the ω was acceptable 0.69. Thus, the “presence” variable consists of six out of seven original items.

### 2.6 Data analysis and statistics

IBM SPSS Statistics (version 27.0) was used for data analysis. An examination of boxplots revealed no outliers to be removed. The central limit theorem ensured that the sampling distribution of the model estimates were normally distributed [53]. The assumption of normality was thus supported. Levene’s test was used to test the assumption of variance. In cases where this assumption was violated, corrected values are reported. A linear mixed-effects ANOVA was used to examine within-group and between group differences in positive affect and negative affect before and after the VR experience by the two VR conditions, assessing hypothesis 1, 2 and 3. To assess hypothesis 4, comparing sense of presence for the desktop VR and immersive VR conditions measured after the VR experience independent samples t-tests were run. Cohen’s [54]guidelines were used when interpreting effect size, i.e. 0.20, 0.50 and 0.80 represent small, medium and large effect sizes.

## 3 Results

All participants were included in the analysis as they all completed the experiment. Table 1 shows the descriptive statistics of the variables in this study.

**Table 1:** Descriptive statistics for study variables.

	Immersive VR (*n* = 69)			Desktop VR (*n* = 65)		
	*M*	*SD*	Range	*M*	*SD*	Range
Age	22.01	3.01	19–40	22.03	2.98	19–32
Previous experience with device	3.04	2.66	0.00–9.00	4.66	3.40	0.00–10.00
PA pre	3.21	0.60	1.90–4.40	2.96	0.59	1.60–4.00
PA post	3.01	0.74	1.70–4.90	2.61	0.77	1.20–4.60
NA pre	1.38	0.37	1.00–2.80	1.35	0.45	1.00–3.20
NA post	2.01	0.82	1.00–3.70	1.98	0.72	1.00–3.70
Sense of presence	7.10	1.25	4.00–9.50	5.77	1.46	1.17–8.83

*Note.* PA = Positive Affect; NA = Negative Affect; M = Mean; SD = Standard Deviation.

### 3.1 Changes in affect before and after the VR experience by condition

Positive affect: We observed a significant linear within-subject effect *F*(1, 132) = 22.30, *p* < 0.001, partial Eta squared = 14, i.e. we observed a reduction in level of positive affect from time 1 (before the VR experience) to time 2 (after the VR experience). Further, there was a significant difference between the two groups, where the immersive VR group had a significant higher level of positive affect *F*(1) = 10.35, *p* = 0.002, partial Eta squared=0.07. There were no significant interaction effect between type of intervention and time on positive affect *F*(1, 132) = 0.1.85, *p* = 0.180.

Negative affect: For negative affect we observed a significant increase in negative affect (main effect) in the sample as a whole *F*(1, 132) = 98.38, *p* < 0.001., partial Eta squared = 43. As with positive affect, there were no significant interaction effect *F*(1, 132) = 0.005, *p* = 0.940. Tests of between-subject effects showed that there was no significant differences between the two groups; *F*(1) = 0.15, *p* = 0.700.

### 3.2 Differences in sense of presence, positive affect and negative affect before and after the VR experience

To test if there were any statistical differences in sense of presence, and previous experience with the device between the immersive VR and desktop VR condition multiple t-tests were conducted (Table 2). We also explored the within- and between effect for positive and negative affect identified in the ANOVA. An alpha level of 0.05 was used for all statistical tests, and Cohen’s *d* was used to examine the magnitude of the effect. On average, the participants that watched the scenario in immersive VR had a higher sense of presence (*M* = 7.10, *SD* = 1.25) than the participants in the desktop VR group (*M* = 5.77, *SD* = 1.46). This difference between the two conditions (*MD* = −1.33) was significant, *t*(132) = −5.70, *p* < 0.001, and large (*d* = 0.99).

**Table 2:** Test of differences between the immersive VR and desktop VR conditions.

	*df*	*MD*	95% CI	*p*	Cohen’s *d*
PEWD*	121.20	1.62	[0.57, 2.67]	0.003	0.53
PA pre	132	−0.25	[−0.45, −0.17]	0.017	0.42
PA post	132	−0.41	[−0.67, −0.15]	0.002	0.54
NA pre	132	−0.03	[−0.17, 0.11]	0.691	0.07
NA post*	131.38	−0.04	[−0.30, 0.22]	0.777	0.05
Sense of presence	132	−1.33	[−1.79, −0.87]	<0.001	0.99

*Note.* PEWD = Previous Experience With Device; PA = Positive Affect; NA = Negative Affect; *df* = Degrees of Freedom; *MD* = Mean Difference; CI = Confidence Interval; * = Corrected for heteroskedasticity.

The participants in the desktop VR group had significantly higher previous experience with the device (*M* = 4.66, *SD* = 3.40) compared to the participants in the immersive VR group (*M* = 3.04, *SD* = 2.66), *t*(121.20) = 3.08, *p =*0.003. There was also a significant higher score of positive affect pre in the immersive VR group (*M* = 3.21, *SD* = 0.60) than the desktop VR group (*M* = 2.96, *SD* = 0.59), *t*(132) = −2.42, *p* = 0.017, which had a small-to-medium affect size (*d* = 0.42). There was no significant difference in negative affect pre between the immersive VR group (*M* = 1.38, *SD* = 0.37) and desktop VR group (*M* = 1.35, *SD* = 0.45), *t*(132) = −0.40, *p* = 0.691 (Table 2). The immersive VR group (*M* = 0.01, *SD* = 0.74) scored significantly higher in positive affect post than the desktop VR (*M* = 2.61, *SD* = 0.77), *t*(132) = −3.12, *p* = 0.002. This difference had a medium effect size (*d* = 0.54). However, there was no significant difference in negative affect post between the immersive VR group (*M* = 2.01, *SD* = 0.82) and the desktop VR group (*M* = 1.98, *SD* = 0.72), *t*(131.38) = −0.28, *p* = 0.778.

We are not aware of any harms or unintended effects in any of the groups, and did not test for e.g. motion sickness in the VR condition.

## 4 Discussion

The aim of this study was to examine whether immersive VR could be a good replacement for desktop VR in terms of soft skills training in higher education by examining affect and presence. This was done through the administration of a virtual scenario of an interpersonal conflict in either immersive VR or desktop VR. The outcomes measured were positive and negative affect, and sense of presence. We found that positive affect was reduced, and negative affect increased after interacting with the VR scenario in both the immersive and desktop versions, however, positive affect was overall greater in the immersive, compared to the desktop version. Moreover, participants had a higher sense of presence in the immersive than in desktop VR. These findings have implications for the use of VR in educational activities, as well as future research.

Multiple important findings were identified with regards to affect. The participants who experienced the scenario through immersive VR had a significantly higher positive affect after playing through the scenario than the participants in the desktop VR group. Thus, supporting Hypothesis 1, stating that there would be greater positive affect after playing through the scenario in immersive VR than in desktop VR. This corresponds with previous findings [e.g. 19, 28] showing that students had higher levels of enjoyment in immersive VR than in desktop VR. In the present study the participants also had higher positive affect prior to start in immersive VR than desktop VR. As the participants were able to identify if they were in the desktop or immersive VR group when answering the affect questions, the high positive affect in the immersive condition may be due to excitement about the immersive VR experience. Positive affect was reduced after watching the conflict scenario in both conditions. This is likely due to the nature of the unpleasant interpersonal conflict theme of the video. Thus, overall, it indicate that the participants felt that the interactive videos affected them emotionally, but they still enjoyed the immersive VR version more than the non-immersive version. However, it is important to note that this could be due to the novelty of the experience with the immersive VR and could be reduced as the technology is maturing. The present study does not clarify this.

The participants also reported low levels of negative affect before and after they played through the scenario in both groups, thus indicating that they did not have a negative attitude either prior to start or after the experiment. However, negative affect increased after watching the scenario, possibly due to the negative content. Results from previous studies imply that learning in immersive VR may have a positive effect on the participants mood with higher registered positive affect in immersive VR than desktop VR [28].

As the scenario dealt with a work-related conflict where the participant played the role of an employee in a first-person perspective, one would assume that such a situation was perceived as challenging and unpleasant for everyone experiencing the scenario. Thus, expecting the scenario itself to have a negative effect on the affective state of the participants. The results reflect this, as the positive affect decreased after experiencing the scenario while negative affect increased in both groups.

Although there was a significant change in affects within both groups, no differences were found in the change of negative affect between the two groups. Thus, Hypotheses 2 and 3, stating that there would be a larger change in positive/negative affect after the scenario in the immersive VR group compared to the desktop VR group, were only supported for positive affect. This is in line with previous studies showing that immersive VR, elicit high levels of sense of presence, also elicit greater emotion than less immersive technology [55].

The participants felt more present in the scenario when watching it through immersive VR, than on a desktop VR, in line with previous research [56], supporting Hypothesis 4. According to the definition of sense of presence, this indicates that when playing through the scenario in immersive VR, the participants felt more as a part of the virtual environment when being physically present in another environment, than the participants in desktop VR.

Overall our study support that level of system immersion, in the present study measured by the type of VR, increase sense of presence and positive affect, which may ultimately influence learning outcomes. This relation is generally accepted in the literature [[57], [58], [59]].

Emotions are shown to influence the learning outcome in multiple ways such as encoding, retrieving, motivation and joy, even though there is inconclusiveness in if this effect has a positive or negative effect on learning outcomes [1]. As positive emotions and enjoyment have been linked to student performance [e.g. 31] simply by being more engaging, the use of immersive VR as an educational technology can promote learning. Thus, both emotion and sense of presence may have an effect on the learning outcomes, as well as influence each other.

### 4.1 Strengths and limitations

Only a few studies found in the literature exploring the effect of immersive VR compare the technology to a control group [4], thus the experimental design with a control group represents a strength of the present study. While we initially planned to implement a cross-lagged design, feedback from the pilot study revealed that it would have compromised the integrity of the results, leading us to have the participants go through only one VR condition each.

Studies in the field of immersive VR are often under-powered and lack a randomized and controlled design with two or more experimental conditions. Thus, the selection of a sample size based on a power analysis performed before the data collection, also strengthens this study. The power analysis resulted in the recruitment of 134 participants, a much larger sample size compared to several previous studies. This is reflected in a review by Checa and Bustillo [4] where the number of participants often were between 20 and 50 participants. A student sample was used in this study, limiting generalisability to the larger population. On the other hand, the VR scenario was developed for use by students during the coursework, and thus has ecological validity.

Studies focusing on immersive VR have often had quite different conditions, e.g., watching an instructional video in desktop VR, while performing the task in immersive VR [9]. This makes it hard to conclude whether the effects observed stem from the immersive VR itself or the performance of the task independent of the immersive VR. In the present study, the two VR scenarios were nearly identical. The similarity in the conditions minimized the number of factors influencing the difference between the two conditions, thus making the outcome of the analysis more valid. However, the novelty of the VR environment can limit the learning experience [60], and probably also their affect and sense of presence and could have influenced the results of the present study.

The scales used in the current study was based on a questionnaire previously used in the measurement of user experience in virtual environments. The questionnaire had a high internal validity and consists of questions from several previously used, well-known questionnaires [49]. As per now, the measurement of soft skills is challenging, and there is no accessible and efficient way of measuring soft skills after an experience in immersive VR [45]. Thus, the present study can provide important knowledge about the effect of immersive VR on affect, which is known to influence learning, but cannot on predict direct learning outcomes. In addition, it is not possible to understand if the VR/desktop differences persist in the long term.

### 4.2 Implications

The findings of this study are relevant for several disciplines such as teaching, learning, and training. Based on previous studies linking sense of presence to higher learning outcomes as previously described [e.g. 19], the results implies that the learning outcome may be higher in the immersive VR condition than for the desktop VR condition [e.g. 4, 14]. Thus, immersive VR seem to be better than desktop VR in eliciting positive emotional responses. As our participants experienced more positive affect before the immersive VR condition compared to the desktop VR condition, immersive VR may be recommended in learning situations where it is important to boost the engagement for the education.

### 4.3 Conclusion

The present study illustrated that students experienced a higher sense of presence in immersive VR as compared to desktop VR when playing through an educational scenario relevant for soft skills. Additionally, they had greater positive affect prior to the experience in immersive VR than desktop VR. This may reflect their motivation or attitude towards the learning, which may influence the learning outcomes. Watching the conflict scenario in immersive and desktop VR reduced positive affect and increased negative affect. However, positive affect was overall greater in the immersive, compared to the desktop version.

The result of the present study supports that immersive VR may replace desktop VR as an educational tool in higher education. This is because immersive VR elicits higher sense of presence than desktop VR. Additionally, students may be more exited to learn through such technology, as reflected by higher levels of positive affect in immersive than desktop scenarios.

Virtual experiences in immersive VR have a notable potential for use in teaching by offering students relevant experiences that they may never be able to experience otherwise. Future research should strive to obtain good measures of the learning effect of soft skill training in immersive VR. More research is needed to investigate the relation between the factors such as immersion, sense of presence and emotion on learning outcomes, and if these influence the immersive VR on specific skills. Longitudinal studies on the effect of long-term use of immersive VR in education is crucial to determine the actual effect of using such technology in educational settings across time. More controlled, randomized studies on large samples, as the present study, are needed to examine the effect immersive VR has on specific learning outcomes and their relations to immersion, sense of presence and emotions. However, the value VR creates for student learning should not be underestimated, and increased focus should be given to this technology and its potential to further enhance teaching excellence in higher education in novel and innovative ways.

## Author contribution statement

Tuva Fjærtoft Lønne: Håvard R Karlsen: Conceived and designed the experiments; Performed the experiments; Analyzed and interpreted the data; Contributed reagents, materials, analysis tools or data; Wrote the paper.

Eva Langvik: Conceived and designed the experiments; Analyzed and interpreted the data.

Ingvild Saksvik-Lehouillier: Conceived and designed the experiments; Analyzed and interpreted the data; Contributed reagents, materials, analysis tools or data; Wrote the paper.

## Data availability statement

Data associated with this study has been deposited at https://doi.org/10.18712/NSD-NSD3101-V1.

## Declaration of competing interest

The authors declare that they have no known competing financial interests or personal relationships that could have appeared to influence the work reported in this paper

## References

[1] Tyng C.M. (2017). The influences of emotion on learning and memory. Front. Psychol..

[2] Ali W. (2020). Online and remote learning in higher education institutes: a necessity in light of COVID-19 pandemic. High Educ. Stud..

[3] Castro R. (2019). Blended learning in higher education: trends and capabilities. Educ. Inf. Technol..

[4] Checa D., Bustillo A. (2020). A review of immersive virtual reality serious games to enhance learning and training. Multimed. Tool. Appl..

[5] Lee E.A.-L., Wong K.W. (2014). Learning with desktop virtual reality: low spatial ability learners are more positively affected. Comput. Educ..

[6] Slater M. (2018). Immersion and the illusion of presence in virtual reality. Br. J. Psychol..

[7] Witmer B.G., Singer M.J. (1998). Measuring presence in virtual environments: a presence questionnaire. Presence Teleoperators Virtual Environ..

[8] Bonde M.T. (2014). Improving biotech education through gamified laboratory simulations. Nat. Biotechnol..

[9] Grassini S., Laumann K., Rasmussen Skogstad M. (2020). The use of virtual reality alone does not promote training performance (but sense of presence does). Front. Psychol..

[10] Makransky G. (2016). Simulation based virtual learning environment in medical genetics counseling: an example of bridging the gap between theory and practice in medical education. BMC Med. Educ..

[11] Radianti J. (2020). A systematic review of immersive virtual reality applications for higher education: design elements, lessons learned, and research agenda. Comput. Educ..

[12] Bowman D.A., McMahan R.P. (2007). Virtual reality: how much immersion is enough?. Computer.

[13] Vesisenaho M. (2019). Virtual reality in education: focus on the role of emotions and physiological reactivity. J. Virtual Worlds Res..

[14] Pellas N., Mystakidis S., Kazanidis I. (2021). Immersive Virtual Reality in K-12 and Higher Education: a systematic review of the last decade scientific literature. Virtual Real..

[15] Thisgaard M., Makransky G. (2017). Virtual learning simulations in high school: effects on cognitive and non-cognitive outcomes and implications on the development of STEM academic and career choice. Front. Psychol..

[16] Moreno R., Mayer R.E. (2002). Learning science in virtual reality multimedia environments: role of methods and media. J. Educ. Psychol..

[17] Stepan K. (2017). Immersive virtual reality as a teaching tool for neuroanatomy. Int. Forum Allergy Rhinol..

[18] Makransky G., Terkildsen T.S., Mayer R.E. (2019). Adding immersive virtual reality to a science lab simulation causes more presence but less learning. Learn. InStruct..

[19] Makransky G., Lilleholt L. (2018). A structural equation modeling investigation of the emotional value of immersive virtual reality in education. Educ. Technol. Res. Dev..

[20] Price M., Anderson P. (2007). The role of presence in virtual reality exposure therapy. J. Anxiety Disord..

[21] Uhm J.-P., Lee H.-W., Han J.-W. (2020). Creating sense of presence in a virtual reality experience: impact on neurophysiological arousal and attitude towards a winter sport. Sport Manag. Rev..

[22] Gross J.J. (2015). Emotion regulation: current status and future prospects. Psychol. Inq..

[23] Vuilleumier P. (2005). How brains beware: neural mechanisms of emotional attention. Trends Cognit. Sci..

[24] Jung N. (2014). How emotions affect logical reasoning: evidence from experiments with mood-manipulated participants, spider phobics, and people with exam anxiety. Front. Psychol..

[25] Phelps E.A. (2004). Human emotion and memory: interactions of the amygdala and hippocampal complex. Curr. Opin. Neurobiol..

[26] Um E. (2012). Emotional design in multimedia learning. J. Educ. Psychol..

[27] Pekrun R. (2006). The control-value theory of achievement emotions: assumptions, corollaries, and implications for educational research and practice. Educ. Psychol. Rev..

[28] Allcoat D., von Mühlenen A. (2018). Learning in virtual reality: effects on performance, emotion and engagement. Res. Learn. Technol..

[29] Picard R.W. (2004). Affective learning — a manifesto. BT Technol. J..

[30] Goetz T. (2006). A hierarchical conceptualization of enjoyment in students. Learn. InStruct..

[31] Valiente C., Swanson J., Eisenberg N. (2012). Linking students' emotions and academic achievement: when and why emotions matter. Child Dev. Perspect..

[32] Jacobson J., Holden L., Kommers P., Richards G. (2005). The Virtual Egyptian Temple.

[33] Kavanagh S. (2017). A systematic review of Virtual Reality in education. Themes Sci. Technol. Educ..

[34] Eckert D., Mower A. (2020).

[35] Pereira O., Costa C.A.A.T. (2017). The importance of soft skills in the university academic curriculum: the perceptions of the students in the new society of knowledge. Int. J. Bus. Soc. Res..

[36] Kim J., Park J.-H., Shin S. (2016). Effectiveness of simulation-based nursing education depending on fidelity: a meta-analysis. BMC Med. Educ..

[37] Plotzky C. (2021). Virtual reality simulations in nurse education: a systematic mapping review. Nurse Educ. Today.

[38] Nyman T.J. (2020). Eyewitness identifications after witnessing threatening and non-threatening scenes in 360-degree virtual reality (or 2D) from first and third person perspectives. PLoS One.

[39] Howard M.C. (2019). Virtual reality interventions for personal development: a meta-analysis of hardware and software. Hum. Comput. Interact..

[40] Manca D., Brambilla S., Colombo S. (2013). Bridging between Virtual Reality and accident simulation for training of process-industry operators. Adv. Eng. Software.

[41] North M.M., North S.M., Coble J.R. (1998). Virtual reality therapy: an effective treatment for the fear of public speaking. Int. J. Virtual Real..

[42] Felnhofer A. (2014). Physical and social presence in collaborative virtual environments: exploring age and gender differences with respect to empathy. Comput. Hum. Behav..

[43] Howard M.C., Gutworth M.B. (2020). A meta-analysis of virtual reality training programs for social skill development. Comput. Educ..

[44] Botke J.A. (2018). Work factors influencing the transfer stages of soft skills training: a literature review. Educ. Res. Rev..

[45] Laker D.R., Powell J.L. (2011). The differences between hard and soft skills and their relative impact on training transfer. Hum. Resour. Dev. Q..

[46] Cohen J. (1988).

[47] Sella F., Raz G., Cohen Kadosh R. (2021). When randomisation is not good enough: matching groups in intervention studies. Psychon. Bull. Rev..

[48] Watson D., Clark L.A., Tellegen A. (1988). Development and validation of brief measures of positive and negative affect: the PANAS scales. J. Pers. Soc. Psychol..

[49] Tcha-Tokey K. (2016).

[50] Hayes A.F., Coutts J.J. (2020). Use omega rather than cronbach’s alpha for estimating reliability. But…. Commun. Methods Meas..

[51] Ding N., Zhou W., Fung A.Y.H. (2018). Emotional effect of cinematic VR compared with traditional 2D film. Telematics Inf..

[52] Kalawsky R.S. (2000).

[53] Pek J., Wong O., Wong A.C.M. (2018). How to address non-normality: a taxonomy of approaches, reviewed, and illustrated. Front. Psychol..

[54] Cohen J. (1992). A power primer. Psychol. Bull..

[55] Diemer J. (2015). The impact of perception and presence on emotional reactions: a review of research in virtual reality. Front. Psychol..

[56] Kim K. (2014). Effects of virtual environment platforms on emotional responses. Comput. Methods Progr. Biomed..

[57] Chittaro L., Buttussi F. (2019). Exploring the use of arcade game elements for attitude change: two studies in the aviation safety domain. Int. J. Hum. Comput. Stud..

[58] Civelek T. (2014). Effects of a haptic augmented simulation on K-12 students' achievement and their attitudes towards physics. Eurasia J. Math. Sci. Technol. Educ..

[59] Nilsson N.C., Nordahl R., Serafin S. (2016). Immersion revisited: a review of existing definitions of immersion and their relation to different theories of presence. Hum. Technol..

[60] Checa D., Miguel-Alonso I., Bustillo A. (2021). Immersive virtual-reality computer-assembly serious game to enhance autonomous learning. Virtual Real.

