# Supplementary material for: The effect of immersion on sense of presence and affect when experiencing an educational scenario in virtual reality: A randomized controlled study
Source: Heliyon. 2023 Jun 12;9(6):e17196. doi: 10.1016/j.heliyon.2023.e17196 (PMC10285157; doi:10.1016/j.heliyon.2023.e17196)
Supplement: Multimedia component 1 [file mmc1.doc]

**
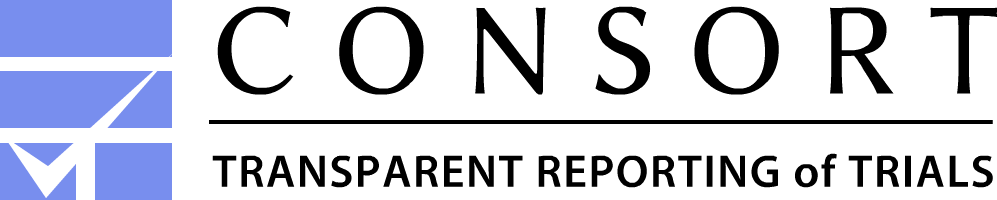
**

**CONSORT 2010 Flow Diagram**

**Allocation**

**Analysis**

**Follow-Up**

**Enrollment**

Assessed for eligibility (n= 134 )

Excluded (n= 0 )

♦  Not meeting inclusion criteria (n= 0 )

♦  Declined to participate (n= 0 )

♦  Other reasons (n= 0 )

Analysed (n= 69 )
♦ Excluded from analysis (give reasons) (n=0)

Lost to follow-up (give reasons) (n=0 )

Discontinued intervention (give reasons) (n= 0)

Allocated to intervention (n=69 )

♦ Received allocated intervention (n= 69 )

♦ Did not receive allocated intervention (give reasons) (n=0 )

Lost to follow-up (give reasons) (n=0 )

Discontinued intervention (give reasons) (n= 0)

Allocated to intervention (n= 65 )

♦ Received allocated intervention (n= 65 )

♦ Did not receive allocated intervention (give reasons) (n= 0 )

Analysed (n= 65)
♦ Excluded from analysis (give reasons) (n=0)

Randomized (n= 134)
