# Supplementary material for: The effect of immersion on sense of presence and affect when experiencing an educational scenario in virtual reality: A randomized controlled study
Source: Heliyon. 2023 Jun 12;9(6):e17196. doi: 10.1016/j.heliyon.2023.e17196 (PMC10285157; doi:10.1016/j.heliyon.2023.e17196)
Supplement: Multimedia component 2 [file mmc2.pdf]

VR-questionnaire English translation

** For peer review only – Unformatted English translation of the original questionnaire – The original questionnaire was adopted to an online system **

Information about consent

This research project is concerned with studying differences between experiencing a virtual environment through VR-glasses and to experience this video format on a normal, flat data screen.

In this questionnaire we ask you to answer thinking of the virtual experience you have just had (VR-glasses or computer). It is important that you think back to how it felt and how you were doing during the experiences and answer as honest as possible.

Completing the questionnaire is assumed to take 7-10 minutes.

Participation is voluntarily. If you want to participate, please press "I want to participate" at the first page of the questionnaire. If you press that you want to participate this is considered consent to participation in the study and storage of your data.

You may whenever you want and without explaining it withdraw your consent. If you wish to withdraw from the study you can demand that we delete all your data, unless we have already anonymized your data or used these in scientific publications.

If you withdraw this will have no negative consequences for you now or later.

If you are a student this study is performed unrelated to your studies at NTNU and will not affect your grades, study credits or other.

Ingvild Saksvik-Lehouillier, department of psychology, NTNU is the project manager.

If you want to withdraw from the study or have questions to the project you can contact project administrator Tuva Fjærtoft Lønne, email: tuvafl@ntnu.no

Todays date: ☐

☐ I want to participate:

Gender:

☐ Woman
☐ Man
☐ Other

Did you participate in the EEG-experiment:

☐ Yes
☐ No

The PANAS

This scale consists of a number of words that describe different feelings and emotions. Read each item and then mark the appropriate answer in the space next to that word. Indicate to what extent [INSERT APPROPRIATE TIME INSTRUCTIONS HERE]. Use the following scale to record your answers.

1	2	3	4	5
very slightly or not at all	a little	moderately	quite a bit	extremely

______ interested	______ irritable
______ distressed	______ alert
______ excited	______ ashamed
______ upset	______ inspired
______ strong	______ nervous
______ guilty	______ determined
______ scared	______ attentive
______ hostile	______ jittery
______ enthusiastic	______ active
______ proud	______ afraid

We have used PANAS with the following time instructions:

Moment	(you feel this way right now, that is, at the present moment)
Today	(you have felt this way today)
Past few days	(you have felt this way during the past few days)
Week	(you have felt this way during the past week)
Past few weeks	(you have felt this way during the past few weeks)
Year	(you have felt this way during the past year)
General	(you generally feel this way, that is, how you feel on the average)

Please stop and watch a video.

The PANAS

This scale consists of a number of words that describe different feelings and emotions. Read each item and then mark the appropriate answer in the space next to that word. Indicate to what extent [INSERT APPROPRIATE TIME INSTRUCTIONS HERE]. Use the following scale to record your answers.

1	2	3	4	5
very slightly or not at all	a little	moderately	quite a bit	extremely

______ interested	______ irritable
______ distressed	______ alert
______ excited	______ ashamed
______ upset	______ inspired
______ strong	______ nervous
______ guilty	______ determined
______ scared	______ attentive
______ hostile	______ jittery
______ enthusiastic	______ active
______ proud	______ afraid

We have used PANAS with the following time instructions:

Moment	(you feel this way right now, that is, at the present moment)
Today	(you have felt this way today)
Past few days	(you have felt this way during the past few days)
Week	(you have felt this way during the past week)
Past few weeks	(you have felt this way during the past few weeks)
Year	(you have felt this way during the past year)
General	(you generally feel this way, that is, how you feel on the average)

Do you have any experience with this type of technology before?

No experience Moderate experience Extensive experience

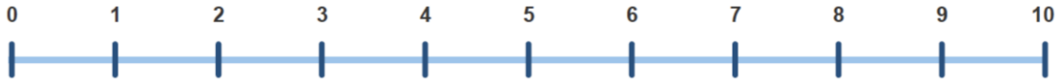

Your virtual experience:

Please think about your experience in the virtual environment.

The virtual environment is the digital environment you have been in during the experience.

You will be presented for claims that you should range on a scale from 1-10 (completely disagree – completely agree) in how well they fit.

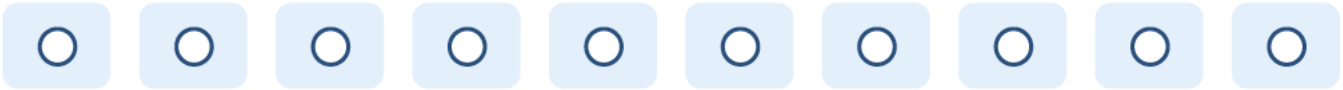

**Items**
1. The virtual environment was responsive to actions that I initiated.
2. My interactions with the virtual environment seemed natural.
3. The visual aspects of the virtual environment involved me.
4. I was able to actively survey the virtual environment using vision.
5. I was able to examine objects closely.
6. I could examine objects from multiple viewpoints.
7. I was involved in the virtual environment experience.
8. I felt proficient in moving and interacting with the virtual environment at the end of the experience.
9. The visual display quality distracted me from performing assigned tasks.
10. I could concentrate on the assigned tasks rather than on the technology.
11. I felt stimulated by the virtual environment.
12. I become so involved in the virtual environment that I was not aware of things happening around me.
13. I identified to the character I played in the virtual environment.
14. I become so involved in the virtual environment that it is if I was inside the game rather than manipulating a gamepad and watching a screen.
15. I become so involved in the virtual environment that I lose all track of time.

**Items** (Continued)
16. I think this technology was easy to use
17. Learning to operate the virtual environment would be easy for me
18. Using this type of technology is a bad idea.
19. Using this type of technology would make my studies more interesting.
20. I would like working with this type of technology
21. I have the resources necessary to use this type of technology
22. The experience in the virtual environment was like being there for real
23. The experience was useful for my learning in work- and organizational psychology

On a scale from 1-10 how did you experience the virtual environment you were in?

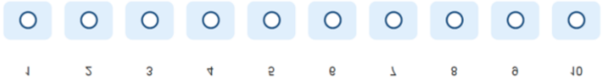

impractical/practical
confusing/clear
unruly/manageable
typical/original
lame/exciting
easy/challenging
amateurish/professional
gaudy/classy
unpresentable/presentable
ugly/beautiful
disagreeable/likeable
discouraging/motivating

Physiological scales

1. I suffered from fatigue during my interaction with the virtual environment.
2. I suffered from headache during my interaction with the virtual environment.
3. I suffered from eyestrain during my interaction with the virtual environment.
4. I felt an increase of my salivation during my interaction with the virtual environment.
5. I felt an increase of my sweat during my interaction with the virtual environment.
6. I suffered from nausea during my interaction with the virtual environment.
7. I suffered from “fullness of the head” during my interaction with the virtual environment.

8. I suffered from dizziness with eye open during my interaction with the virtual environment.

All answered on a scale from 1 (not at all) – 5 (moderate) – 10 very much

1 Ikke i det hele tatt	2	3	4	5 Moderat	6	7	8	9	10 I svært stor grad
○	○	○	○	○	○	○	○	○	○

What do you think was the most positive with the virtual experience?

What was the most negative with the virtual experience?

Do you have any suggestions to improving the virtual experience?
